# Gestational bisphenol A exposure alters energy homeostasis and adult hypothalamic neurogenesis in female mice

**DOI:** 10.1038/s41598-024-66726-2

**Published:** 2024-07-12

**Authors:** Kira M. Feighan, Dinushan Nesan, Deborah M. Kurrasch

**Affiliations:** 1 Department of Medical Genetics, Cumming School of Medicine, University of Calgary, Calgary, AB Canada; 2 Alberta Children’s Hospital Research Institute, University of Calgary, Calgary, AB Canada; 3 Hotchkiss Brain Institute, University of Calgary, Calgary, AB Canada; 4 Present Address: Montreal Neurological Institute, McGill University, Montreal, QC Canada

**Keywords:** Endocrinology, Hypothalamus, Adult neurogenesis

## Abstract

Regulation of physiological homeostasis, including energy balance, is thought to be modified by low levels of adult neurogenesis in the hypothalamus. Hormones such as oestradiol can influence both embryonic and adult hypothalamic neurogenic programs, demonstrating a sensitivity of hypothalamic neural progenitor cells to endogenous hormones. Previously we showed that gestational exposure to environmental levels of the xenoestrogen bisphenol A (BPA) changed neural progenitor cell behaviors in the embryo; however, we did not examine if these changes were permanent to affect adult neurogenesis. Here we investigated whether adult neuro- and/or gliogenesis were altered in mice prenatally exposed to BPA and placed on a high-fat diet challenge. Gestationally exposed adult female mice on a standard diet gained less weight than non-BPA controls, whereas gestationally exposed BPA females on a high-fat diet gained more weight than controls. Males exposed to gestational BPA showed no differences in weight gain relative to control males. Concomitantly, adult neurogenesis was increased in the VMH, DMH, and PVN of adult female mice exposed to BPA on standard diet, suggesting that disrupted adult neurogenesis might perturb normal energy balance regulation in females. These results add to growing evidence that low-dose BPA exposure in utero causes changes to adult hypothalamic function.

## Introduction

Bisphenol A (BPA) is a xenoestrogen and a widely studied endocrine disrupting plasticizer^1,2^. This environmental contaminant has agonistic action at estrogen and androgen receptors as well as antagonistic action at androgen and thyroid receptors, with proposed impacts at glucocorticoid receptors^1^. Although many governmental regulatory bodies deem BPA safe at current environmental levels, the compound exhibits a non-monotonic dose–response curve and adverse effects are observed at the lowest tested doses^3–6^. Developmental exposure to BPA in humans is correlated with a range of neurodevelopmental disorders^7^; however, the mechanisms underlying the behavioural phenotypes of gestational BPA exposure remain poorly understood.

The neuroendocrine hypothalamus is a critical system for regulating physiological homeostasis, serving as a regulatory control center^8,9^. The collection of neuropeptidergic neurons and non-endocrine neurons across the dozen distinct hypothalamic nuclei contribute to the regulation of energy metabolism, circadian rhythms, reproduction, temperature, and fluid balance, among other physiologies. We previously found in zebrafish that embryonic BPA exposure accelerates developmental neurogenesis throughout the hypothalamus via agonism of androgen and estrogen receptors^10^, as well as alterations in the spatial localization and specification of suprachiasmatic nucleus neurons, causing disrupted circadian rhythms in mice^11^. These changes to embryonic neurogenesis suggest that gestational BPA exposure may cause lasting effects on hypothalamic neural stem populations that will impact adult hypothalamic neurogenesis as well.

Although historically the hypothalamus was thought to remain relatively static throughout adulthood, emerging evidence shows that low levels of adult neurogenesis contribute to various hypothalamic nuclei^12,13^. The identity of neural progenitor cells remains debated; however, the neurogenic niche region is generally considered to include the hypothalamic ventricular zone at the level of the paraventricular, ventromedial, and arcuate nuclei, as well as the median eminence^14^. Adult hypothalamic neurogenesis is best studied for its influence on energy homeostasis, but it is also implicated in reproduction, exercise, and temperature control^13,15^. Cellular proliferation in the adult hypothalamus can be stimulated by a variety of factors, including growth factors such as ciliary neurotrophic factor (CNTF) and insulin-like growth factor 1 (IGF-1), or changes to energy balance such as those caused by a high-fat diet^15–18^. Different stimulants can have varied effects on the hypothalamus, which are dependent on length of exposure and are often sexually dimorphic. For example, one month on a high-fat diet induces adult neurogenesis in the median eminence of female mice, whereas mice fed a high-fat diet longer than a month show decreased proliferative capacity^17,18^. The sexually dimorphic effects of some stimulants indicate a potential role for sex hormones to influence adult hypothalamic neurogenesis^19–21^. Indeed, oestradiol exposure protects against obesity and inhibits adult hypothalamic neurogenesis in ovariectomized female mice fed a high-fat diet, and increases adult hypothalamic neurogenesis in female mice fed a standard diet^22^.

Here, we investigated the sensitivity of adult hypothalamic neurogenesis to gestational BPA exposure, and the interaction of BPA exposure with a challenge of high-fat diet later in life. We showed that BPA alters weight gain in adolescents and adult females, interacting with diet treatment to determine weight gain or loss. Moreover, we found that BPA increased adult hypothalamic neurogenesis in the ventromedial hypothalamus (VMH), paraventricular nucleus (PVN), and dorsomedial hypothalamus (DMH) of female mice at an early timepoint, but that these changes are not detected three weeks later. These findings reveal a role for gestational BPA exposure in energy balance disruptions and demonstrate sexually dimorphic effects on adult hypothalamic neurogenesis.

## Results

### Gestational BPA exposure disrupts weight gain

#### Adolescents

**Figure 1:**
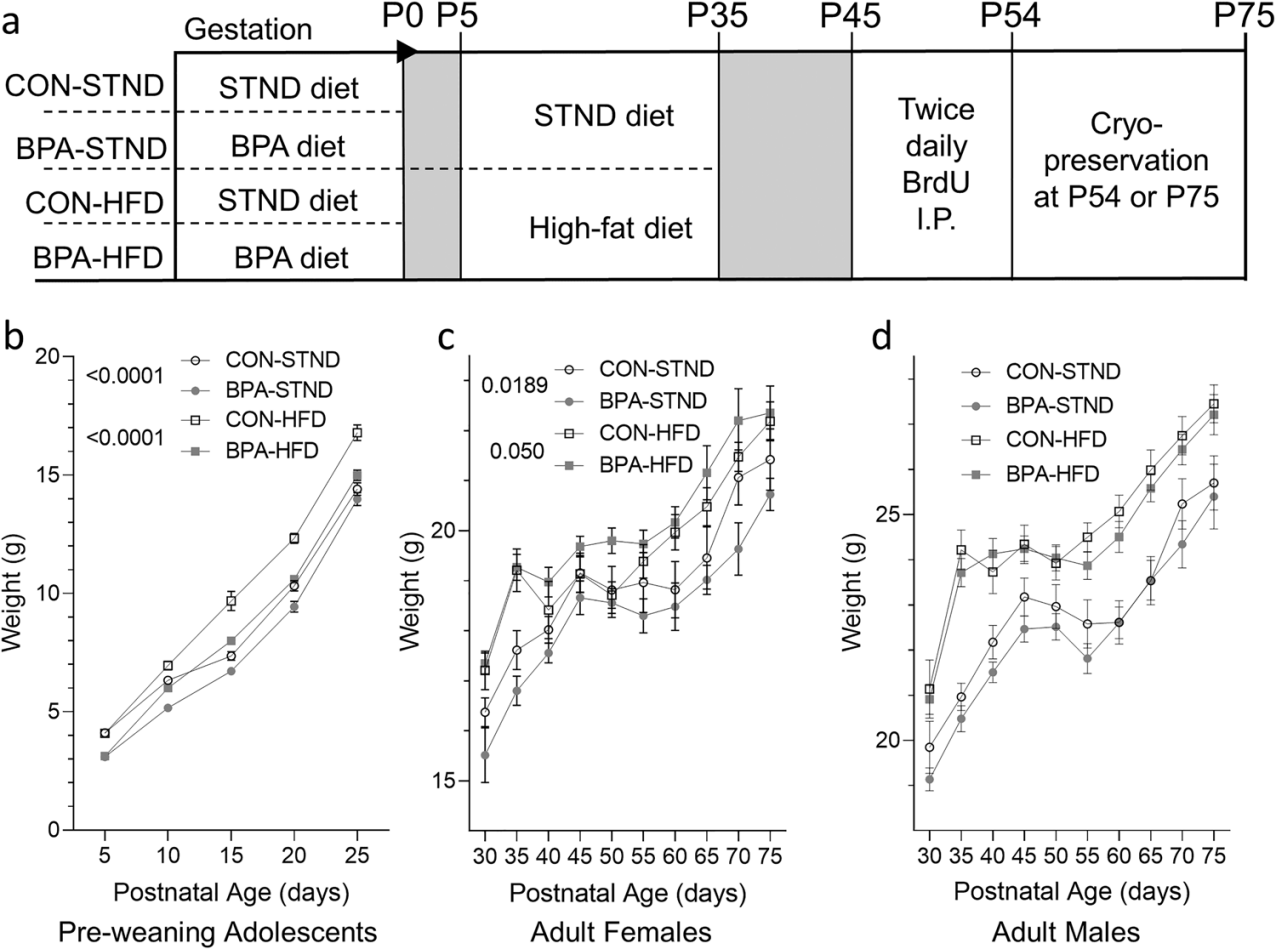
Weight gain in animals exposed to BPA during gestation is different from controls. (**a**) Experimental paradigm. (**b**) Weights of adolescent mice prior to weaning (P5-25). Animals exposed to BPA during gestation gained less weight than control animals in both the STND (*p* < 0.0001) and HFD (*p* < 0.0001) conditions (N = 4–6 per group, n = 33–59 per group). (**c**) Weights of post-weaning adult females (P25-75). Animals exposed to BPA during gestation gained less weight in the STND condition (*p* = 0.0189); however gained more weight in the HFD condition (*p* = 0.050) (N = 4–6 per group, n = 10–23 per group). (**d**) Weights of post-weaning adult males (P25-75). No difference in weight gain was observed between groups (N = 4–6 per group, n = 16–36 per group). (All data is presented as mean ± SEM, two-way ANOVA between groups with Tukey post-hoc test for pairwise comparisons).

To examine the physiological effects of prenatal BPA exposure followed by a high-fat diet challenge later in life, BPA or control animals were placed on a high-fat diet from P5-35 and compared to those maintained on a standard chow (referred to as STND, experimental timeline in Fig. 1a). All animals were weighed every five days starting at P5 through P25. Males and females were indistinguishable prior to weaning, so sex was not considered. A two-way ANOVA test was used to analyze postnatal weight, with Tukey post-hoc test if significant main effects were determined (F (12, 746) = 4.041). Control pups on a high-fat diet gained the most weight (n = 42, N = 5, mean = 9.965, SD = 4.892), with animals exposed to BPA in utero on a high-fat diet (n = 59, N = 6, mean = 8.541, SD = 4.520) weighing statistically less (*p* < 0.0001; Fig. 1b). For animals on a standard diet, the control (n = 33, N = 4, mean = 8.503, SD = 3.983) weighed more than the gestationally-exposed BPA animals (n = 41, N = 5, mean = 7.768, SD = 4.219) on this same chow (*p* < 0.0001; Fig. 1b).

#### Adult females

The weights of post-weaning adult female mice (P30-75) were measured every five days and analyzed with a two-way ANOVA and Tukey post-hoc test (F (27, 454) = 0.6563). In this group, a split response was observed. Specifically, females gestationally exposed to BPA placed on the standard diet (n = 10, N = 5, mean = 18.33, SD = 1.452) gained less weight than control animals (n = 17, N = 4, mean = 18.97, SD = 1.498) on this diet (*p* = 0.0189; Fig. 1c), whereas BPA females on the HFD (n = 23, N = 6, mean = 20.07, SD = 1.511) gained more weight than control animals (n = 16, N = 5, mean = 19.62, SD = 1.470) on this diet (*p* = 0.050; Fig. 1c).

#### Adult males

The weights of post-weaning adult male mice (P30-75) were measured every five days and analyzed with a two-way ANOVA (F (27, 778) = 0.9354). Males gestationally exposed to BPA were not statistically different from controls in either the standard or high-fat diet group (Fig. 1d).

### Gestational BPA exposure affects adult neurogenesis but not adult gliogenesis

#### Neurogenesis

**Figure 2:**
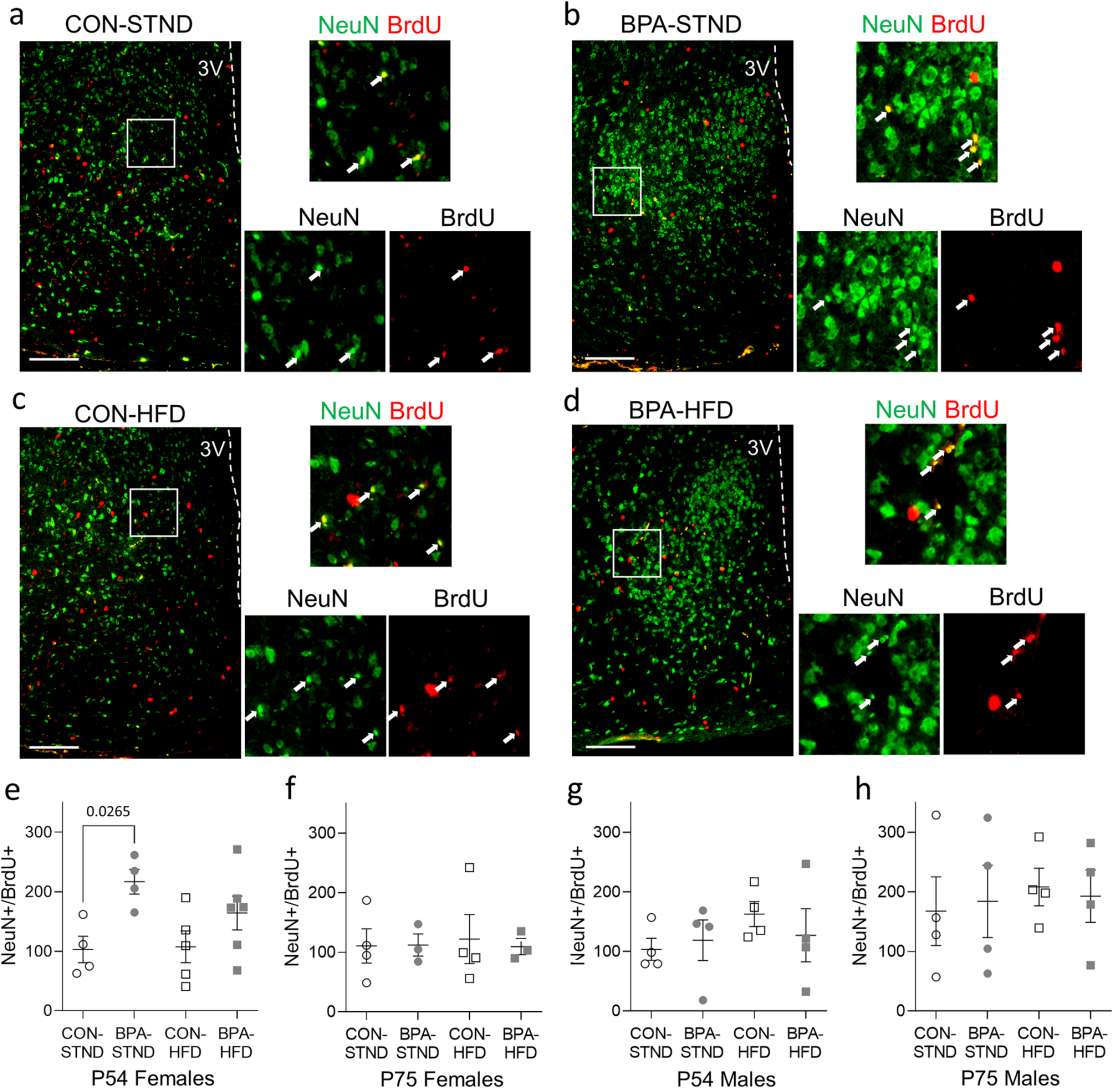
Gestational BPA exposure increases neurogenesis in P54 adult females. (**a**–**d**) Representative images of the female hypothalamus at P54 in each condition. Co-labelled cells for NeuN and BrdU are indicated with arrows. (**e**) Total NeuN^+^/BrdU^+^ co-labelled cells in the female hypothalamus at P54. Animals exposed to BPA during gestation in the STND condition had increased levels of double positive cells in the hypothalamus relative to controls (*p* = 0.0265, N = 4 per group, n = 4 per group). There was no difference in double positive cells between BPA-exposed and control animals in the HFD condition (*p* = 0.2309, N = 5–6 per group, n = 5–6 per group). (**f**) Total NeuN^+^/BrdU^+^ co-labelled cells in the female hypothalamus at P75. No difference in double positive cells was observed between BPA-exposed and control animals in either STND or HFD condition (N = 3–4 per group, n = 3–4 per group). (**g**) Total NeuN^+^/BrdU^+^ co-labelled cells in the male hypothalamus at P54. No difference in double positive cells was observed between BPA-exposed and control animals in either STND or HFD condition (N = 4 per group, n = 4 per group). (**h**) Total NeuN^+^/BrdU^+^ co-labelled cells in the male hypothalamus at P75. No difference in double positive cells was observed between BPA-exposed and control animals in either STND or HFD condition (N = 4 per group, n = 4 per group). (All data is presented as mean ± SEM, one-way ANOVA with Sidak’s post-hoc test for pairwise comparisons. Scale bar, 100 µm).

To determine if this disruption in weight gain in BPA-exposed animals associates with changes in adult neurogenesis, we conducted birthdating experiments in mice fed standard chow or challenged with a high fat diet (P5-P35), following a paradigm shown to stimulate adult neurogenesis^16^. All animal groups were injected intraperitoneally (i.p.) with BrdU (50 mg/kg bodyweight) twice a day (11 am and 8 pm) for nine days from P45 to P53. A subset of each litter was sacrificed at P54 to capture the number of neurons born within this nine day window and the remaining animals were sacrificed at P75 to determine the number of neurons still present two weeks later (experimental timeline in Fig. 1a). The number of cells co-labelled for BrdU and the neuronal marker NeuN in each group were quantified throughout the hypothalamus and compared using a one-way ANOVA with Sidak’s post-hoc test for pairwise comparisons. BPA-exposed females on standard diet at P54 (n = 4, mean = 216.9, SD = 41.32) displayed an increase in neuronal birth relative to control animals (n = 4, mean = 102.9, SD = 44.50) (F (3, 15) = 3.724, *p* = 0.0265; representative images Figs. 2a–d,e), which notably is the group that gained the least weight during adolescence and adulthood (Fig. 1b,c). In P54 females on high-fat diet (Fig. 2e) or P75 females on either diet (Fig. 2f) no difference in neurogenesis was observed between control and BPA-exposed animals. In males at P54 and P75, no difference in neuronal birth was observed between control and animals exposed gestationally to BPA on either diet (Fig. 2g,h).

#### Gliogenesis

**Figure 3:**
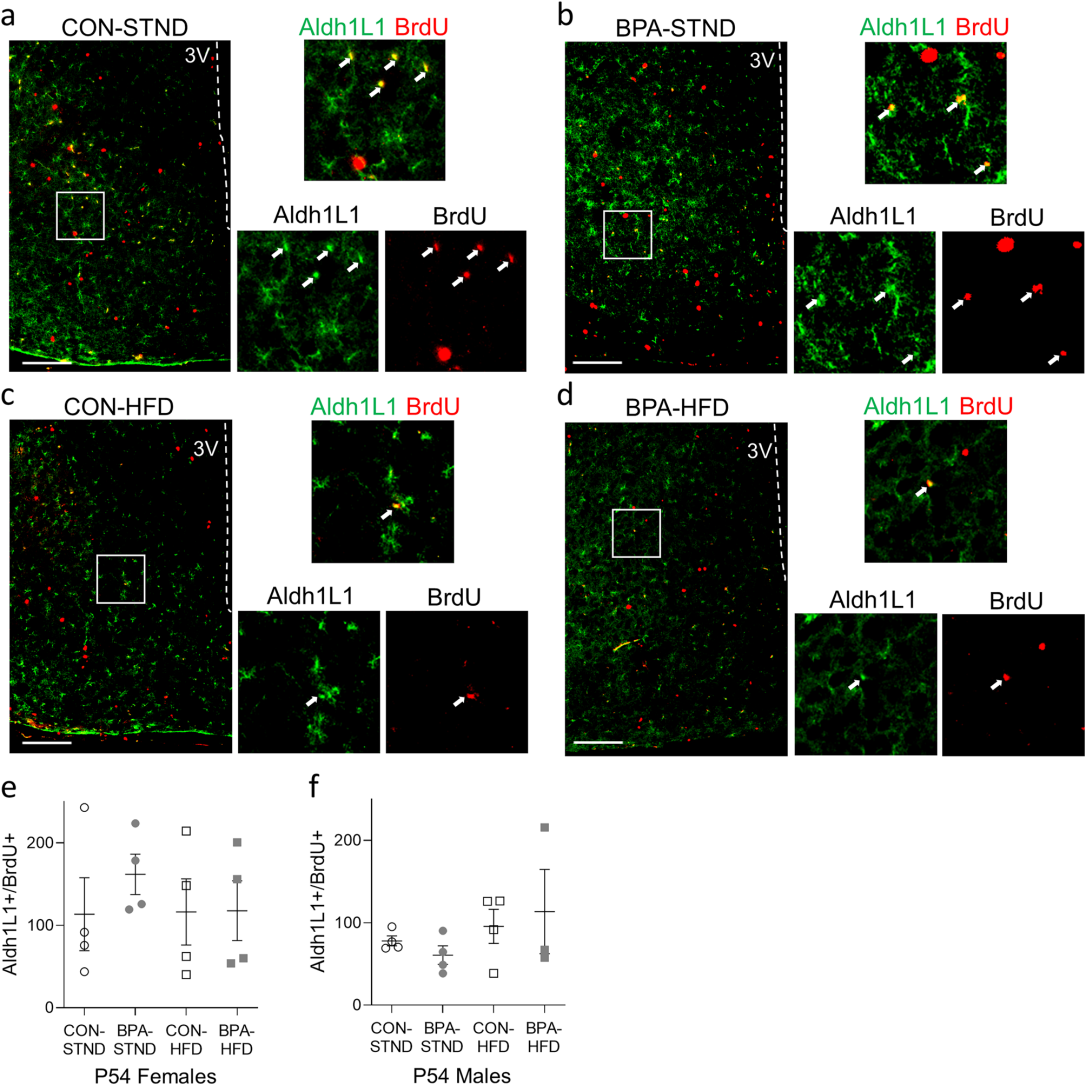
Gestational BPA exposure has no impact on adult astrocyte gliogenesis. (**a**–**d**) Representative images of the female hypothalamus at P54 in each condition. Co-labelled cells for Aldh1L1 and BrdU are indicated with arrows. (**e**, **f**) Total Aldh1L1^+^/BrdU^+^ co-labelled cells in the female and male hypothalamus at P54. No difference in double positive cells was observed between BPA-exposed and control animals in either STND or HFD condition (N = 3–4 per group, n = 3–4 per group). (All data is presented as mean ± SEM, one-way ANOVA. Scale bar, 100 µm).

**Figure 4:**
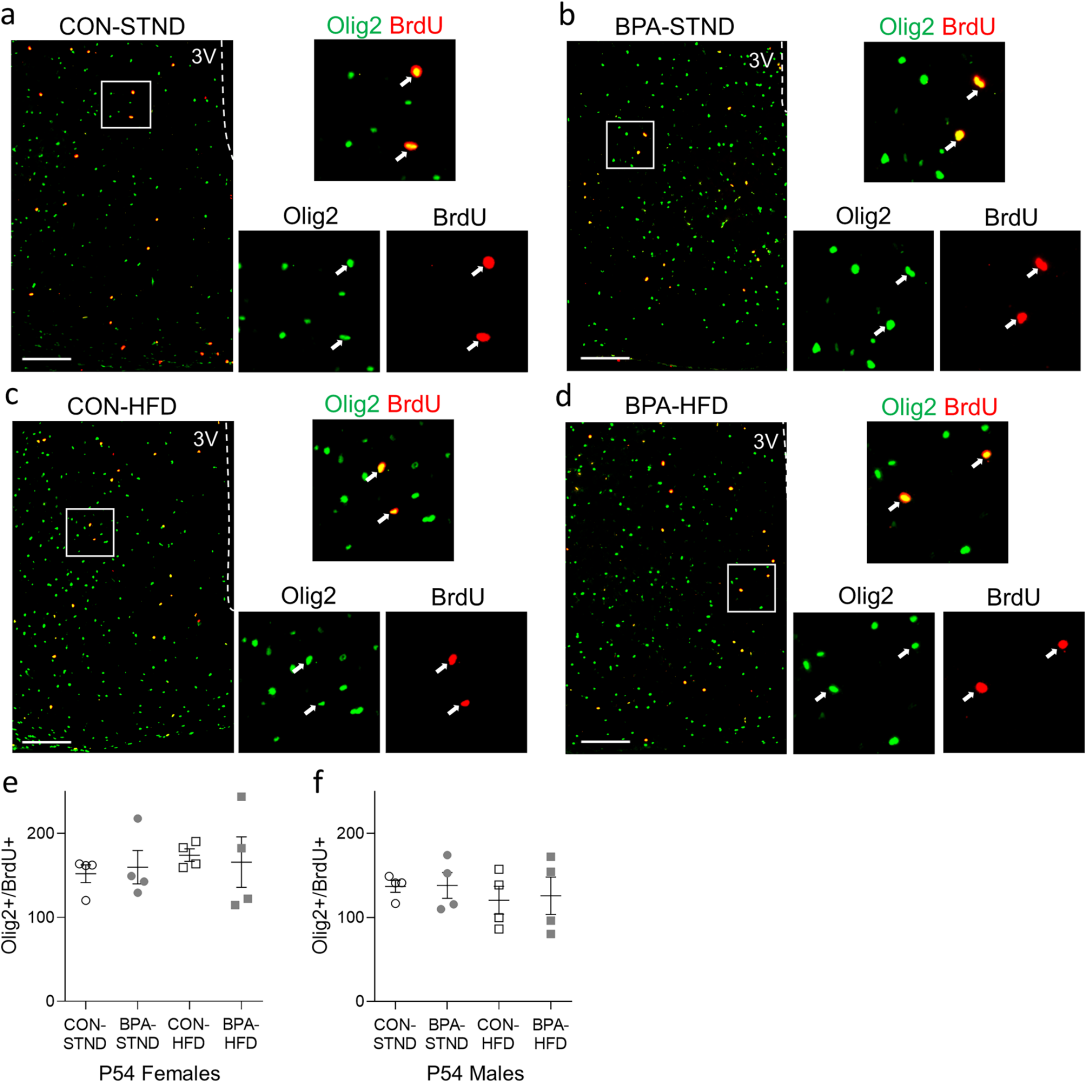
Gestational BPA exposure has no impact on adult oligodendrogenesis. (**a**–**d**) Representative images of the female hypothalamus at P54 in each condition. Co-labelled cells for Olig2 and BrdU are indicated with arrows. (**e**, **f**) Total Olig2^+^/BrdU^+^ co-labelled cells in the female and male hypothalamus at P54. No difference in double positive cells was observed between BPA-exposed and control animals in either STND or HFD condition (N = 3–4 per group, n = 3–4 per group). (All data is presented as mean ± SEM, one-way ANOVA. Scale bar, 100 µm).

Since hypothalamic neural progenitors also can give rise to astrocytes and oligodendrocytes, changes in gliogenesis in the hypothalamus were analyzed as per above at P54. Cells co-labelled for BrdU^+^/Aldh1L1^+^ (astrocytes; representative images in Fig. 3a–d) or BrdU^+^/Olig2^+^ (oligodendrocytes; representative images in Fig. 4a–d) were compared using a one-way ANOVA. No significant difference in total number of BrdU^+^/Aldh1L1^+^ cells or total number of BrdU^+^/Olig2^+^ cells was observed between control and BPA conditions on either diet in males or females (Figs. 3e,f, Fig. 4e,f).

### Adult neurogenesis in the tuberal hypothalamus is sensitive to gestational BPA exposure

**Figure 5:**
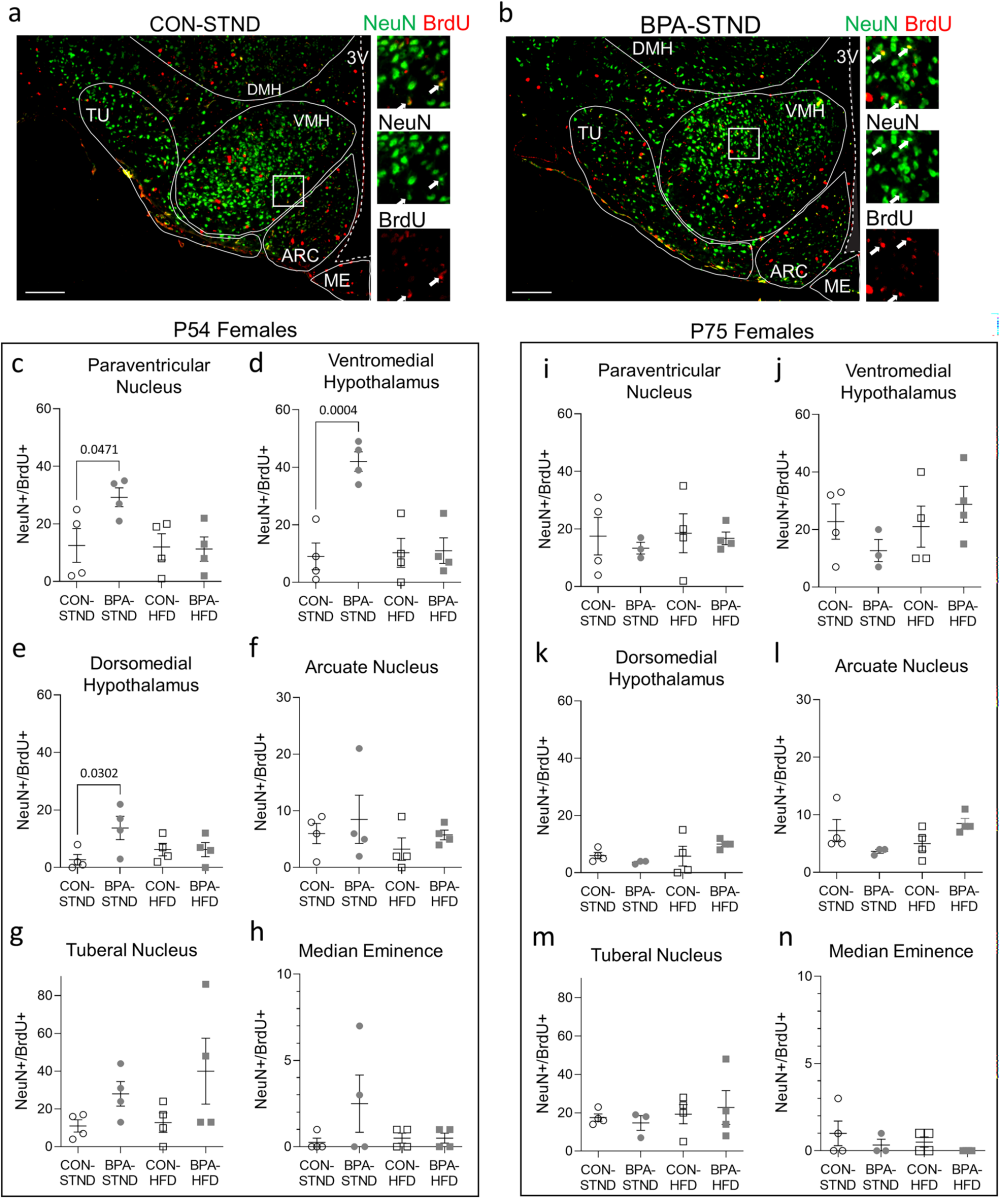
Gestational BPA exposure increases neurogenesis in specific nuclei of the tuberal hypothalamus in adult females at P54. (**a**, **b**) Representative images of the female hypothalamus at P54 in the CON-STND and BPA-STND conditions. Approximate locations of the dorsomedial hypothalamus (DMH), ventromedial hypothalamus (VMH), tuberal nucleus (TU), arcuate nucleus (ARC), and median eminence (ME) are indicated. Co-labelled cells for NeuN and BrdU are indicated with arrows. (**c**–**h**) Total NeuN^+^/BrdU^+^ co-labelled cells in the female hypothalamus at P54 in the paraventricular nucleus, ventromedial hypothalamus, dorsomedial hypothalamus, arcuate nucleus, tuberal nucleus, and median eminence. Animals exposed to BPA during gestation in the STND condition had increased counts of double positive cells in the paraventricular nucleus (*p* = 0.0471), the ventromedial hypothalamus (*p* = 0.0004), and the dorsomedial hypothalamus (*p* = 0.0302) (N = 4 per group, n = 4 per group). No difference in double positive cells was observed between BPA-exposed and control animals in the HFD condition in these regions, or the other regions examined (N = 4 per group, n = 4 per group). (**i**–**n**) Total NeuN^+^/BrdU^+^ co-labelled cells in the female hypothalamus at P75 in the paraventricular nucleus, ventromedial hypothalamus, dorsomedial hypothalamus, arcuate nucleus, tuberal nucleus, and median eminence. No difference in double positive cells was observed between BPA-exposed and control animals in either STND or HFD condition in any of the regions examined (N = 3–4 per group, n = 3–4 per group). (All data is presented as mean ± SEM, one-way ANOVA with Sidak’s post-hoc test for pairwise comparisons. Scale bar, 100 µm).

To determine whether the observed changes in adult neurogenesis were localized to a specific hypothalamic nucleus, one-way ANOVA with Sidak’s post-hoc test for pairwise comparisons were used to compare BrdU^+^/NeuN^+^ cells between female groups in the paraventricular nucleus (PVN), ventromedial hypothalamus (VMH), arcuate nucleus (ARC), tuberal nucleus (TU), dorsomedial hypothalamus (DMH), median eminence (ME) (representative images in Fig. 5a,b), as well as the medial preoptic nucleus (MPO), suprachiasmatic nucleus (SCN), and anterior hypothalamic nucleus (AHN) as demarked by NeuN staining to define the cell body-dense nuclear regions (as per Allen Brain Map) (data not shown). The number of double positive cells varied across regions, with the ME particularly low. Females at P54 on standard diet gestationally exposed to BPA showed increased neuronal birth in the PVN (F (3, 12) = 3.595, CON-STND: n = 4, mean = 12.50, SD = 11.73; BPA-STND: n = 4, mean = 29.25, SD = 6.551; *p* = 0.0471; Fig. 5c), the VMH (F (3, 12) = 13.06, CON-STND: n = 4, mean = 9.00, SD = 9.274; BPA-STND: n = 4, mean = 42.00, SD = 6.782; *p* = 0.0004; Fig. 5d), and the DMH (F (3, 12) = 2.843, CON-STND: n = 4, mean = 2.750, SD = 3.594; BPA-STND: n = 4, mean = 13.75, SD = 8.057; *p* = 0.0302; Fig. 5e), with no differences in the other regions examined (Fig. 5e–h). No differences in adult neurogenesis were observed at P54 between female controls and BPA-exposed on a high-fat diet for any of the regions examined (Fig. 5 c-h). Likewise, P75 females showed no difference in neurogenesis between control and gestational BPA exposed groups on either diet in any of the regions examined (Fig. 5i-n).

## Discussion

In the present study we examined the effects of gestational low-dose BPA exposure on adult neurogenesis in the hypothalamus and any interaction with energy-balance disruption via adolescent high-fat diet exposure. We found that gestational BPA exposure disrupts weight gain in a sexually dimorphic manner dependent on age and diet treatment. Specifically, gestational BPA exposure decreased weight gain in adolescent mice on both diets, and in adult female mice on the standard diet; however increased weight gain was observed in adult female mice on a high-fat diet. No effects were observed in adult males. Additionally, we found that gestational BPA exposure increased adult neurogenesis in the female hypothalamus on standard diet when measured immediately after the BrdU injection period. However, this increase in neurogenesis was absent when measured three weeks following the injection period, suggesting perhaps that these newly born neurons were only transiently required. These findings suggest that gestational exposure to BPA causes lasting changes in progenitor behaviors that underlie adult hypothalamic neurogenesis and perhaps leads to effects on energy balance regulation.

As an endocrine disrupting compound with effects on the hypothalamus, BPA has previously been implicated in disruptions of energy balance and metabolism^23^. BPA is classically considered an obesogen; however, this finding remains controversial and is dependent on experimental conditions, perhaps due to the pleiotropic effects of BPA^24^. The obesogenic characterization of BPA directly contradicts the weight loss seen in adolescents and adult females on standard diet in this study. Other experiments show similar deviations from this characterization based on factors such as sex, diet treatment, window of BPA exposure, and age of outcome^24^. BPA has sexually dimorphic effects on energy balance and females typically display greater alterations in weight gain resulting from BPA exposure than males^25,26^. This dimorphism may have contributed to the lack of phenotype in BPA-exposed adult males. Diet has important modulatory effects on the ability of BPA to act as an obesogen^24^. High-fat diet is known to enhance the obesogenic properties of BPA, a factor that likely contributed to the weight increase observed in adult females on high-fat diet^26^. This effect is often restricted to females, since studies show that high-fat diet does not induce more weight gain in BPA-exposed males than control males, potentially due to a ceiling effect^27^. In a study comparing different exposure windows, prenatal exposure from gestational day six to birth resulted in decreased weight of young mice, whereas postnatal exposure had the opposite effect^28^. Thus, the timing of gestational exposure used here may have contributed to some of the decreases in weight. The effect of the age at which outcomes are measured is less apparent since some studies show a decrease in weight during adolescence compared to adulthood, while other studies show the opposite^26,29^. However, it is evident that energy balance processes are differentially impacted in adolescents compared to adults, which may have contributed to the robust weight decrease in adolescents that in adults was only observed in females on standard diet. It should also be noted that while the differences in weight gain observed in females between the control and BPA groups on standard diet were significant, this effect was relatively small and may have arisen from a difference in power due to the discrepancy in group sizes. Further research and replication will be required to validate these findings.

Beyond energy homeostasis disruptions, we report an increase in adult neurogenesis in the VMH, DMH, and PVN of female mice immediately following the injection period at P54. The VMH is involved in energy homeostasis and feeding behaviour, with developmental disruptions to the VMH causing obesity in adulthood^30^. This region is also implicated in female sexual behaviour and cardiovascular function. The DMH plays an important role in maintaining energy homeostasis through influence on feeding behaviour and energy expenditure^31^. Similar increases in adult neurogenesis in the VMH and DMH were reported previously in studies focused on the role of adult neurogenesis in energy homeostasis. Weight loss resulting from either exposure to estrogen in ovariectomized mice or to CNTF is associated with an increase in adult neurogenesis in the VMH, DMH and ARC^15,22^. Interestingly, each of these studies showed an increase in adult neurogenesis in the ARC, another critical energy balance control region^32^, an effect that was not seen following the gestational BPA exposure in this study. However, the present study did show an increase in adult neurogenesis in the PVN, a region that receives extensive neuronal projections from the ARC^18^. The paraventricular nucleus is a significant autonomic control center with a wide range of outputs that regulate energy balance alongside growth, stress, and reproduction, among others^18,33^. Increased adult neurogenesis in the PVN is observed following BDNF infusion into the lateral ventricles of rats and decreased adult neurogenesis is observed in female, but not male, rats following pre-pubertal gonadectomy^34,35^. Our study here adds to growing evidence that increased adult neurogenesis in response to energy balance regulation is associated with weight loss. Furthermore, the specific alterations to adult neurogenesis in energy homeostatic regions suggests that the induction of adult hypothalamic neurogenesis via gestational BPA exposure may be one mechanism by which BPA alters energy homeostasis. More research is needed to determine if other behavioural outputs of these regions are impacted, such as stress, temperature homeostasis, or reproductive behaviour.

Given the xenoestrogenic properties of BPA, it is likely that an estrogenic mechanism of action underlies the changes in adult neurogenesis observed in gestationally BPA-exposed females. Indeed, the increase in adult neurogenesis and weight loss in adult females is strikingly similar to the effects observed in ovariectomized mice given estrogen^22^. Estrogens are traditionally involved in the maintenance of energy homeostasis through regulation of lipid and glucose metabolism, and in humans, the absence of estrogen resulting from menopause is correlated with obesity^36^. Action at the androgen receptors perhaps is a less likely candidate since no differences in weight were observed in adult males, and existing correlations between obesity and androgen levels suggest that androgen deficiency is a result of rather than a cause of obesity^37,38^. Action at thyroid receptors cannot be precluded as a potential contributing factor since thyroid status is closely linked with weight changes and energy homeostasis^39^. Thus, the alterations to weight gain and adult neurogenesis observed in this experiment were perhaps due to a permanent disruption in estrogen signaling resulting from gestational BPA exposure, with potential modulation via thyroid receptor antagonism also worth considering.

These changes in adult neurogenesis were only observed in females on a standard diet at P54 immediately following the nine-day BrdU injection period, with no changes in adult neurogenesis detected in this same group when measured at P75, three weeks following the injection period. Programmed cell death (PCD) in the BPA exposed group at P75 might explain the loss of an effect as perhaps these newly born neurons failed to survive. PCD is a critical process for the regulation of adult neurogenesis throughout the brain; however the study of PCD in relation to adult neurogenesis in the hypothalamus remains limited^40^. More research is required to determine if PCD is the mechanism behind this effect, and what, if any, impact this process has on energy balance.

Despite this hypothesized relationship between energy balance disruptions and BPA-induced alterations to adult hypothalamic neurogenesis, there are several other mechanisms by which BPA exposure could disrupt this homeostasis^23^. Neonatal exposure to BPA is known to downregulate estrogen receptor expression in the arcuate nucleus, the key hypothalamic region for the integration of hunger and satiety signals^41,42^. Neonatal and perinatal action of BPA at estrogen receptors in pancreatic β-cells results in increased insulin synthesis and release, and increased leptin levels, contributing to the eventual development of insulin resistance and impaired glucose tolerance^43–46^. BPA is commonly associated with the induction of hyperactivity^24^. Hyperactivity has been observed in both males and females under a variety of conditions of BPA exposure, including the dose and gestational timing used in this experiment, and typically results in decreased weight despite increased caloric intake^11,47^. The present study did not provide any means to evaluate the contribution of other pathways towards the alteration of energy homeostasis, and as such it cannot be concluded at this point whether the observed changes in energy balance following gestational BPA exposure are the result of changes to adult hypothalamic neurogenesis, some other effect of BPA, or a combination of both.

Here, we demonstrate a role for gestational BPA exposure in disruptions of energy homeostasis in adolescents and adult females and suggest links to adult hypothalamic neurogenesis. Increased adult neurogenesis in energy control centers of the female hypothalamus is closely associated with the observed weight loss and is likely correlated with an estrogenic mechanism of action. These disruptions were observed at a dose significantly lower than commonly reported values in human newborn umbilical cord blood^48,49^. This adds to a growing body of evidence demonstrating that low-dose BPA exposure adversely effects multiple organ systems, especially the brain^3,50,51^.

## Methods

### Animal husbandry

All experimental and animal protocols were approved by the University of Calgary Animal Care Committee, followed the Guidelines for the Canadian Council of Animal Care, and followed the ARRIVE guidelines for animal research^52^. Mice were housed at the Health Sciences Animal Resource Center at the University of Calgary, had ad libitum access to water, and fed lab standard diet (LabDiet Pico-Vac Lab Rodent Diet 5061), except during gestation or adolescence as stated. Wild-type C57Bl6 mice from Charles River were paired and checked daily for breeding success. Upon positive plug, pregnant dams were placed on either a control diet (7% corn oil, Envigo diet code TD.120176) or a BPA diet (50 μg/kg BPA, Envigo diet code TD.160491, as per Nesan et al., 2021). Dams were returned to a standard lab diet after pups were born. At P21 pups were weaned, separated by sex, and placed in new cages. A subset of the control and BPA litters was placed on a high-fat diet (60% calories as fat, Research Diets #D12492) from P5 to P35, with the matching diet also fed to mothers prior to weaning. Animals were not randomized to their treatment groups as treatment was gestational and thus applied to entire litters. Dams were used for 1–3 rounds of breeding and remained in the same diet group each time, with rest periods on standard lab diet for 6–8 weeks following weaning. All mice were weighed every five days from P5 to P75. Intraperitoneal (IP) injections of 5-bromo-2’-deoxyuridine (BrdU) at 50 mg/kg bodyweight were conducted twice a day (11am and 8 pm) for nine days from P45 to P53 on all groups. A subset of each litter was sacrificed at P54, and the remaining were sacrificed at P75.

### Immunohistochemistry

On P54 or P75, adult animals were anaesthetized using isoflurane and perfused with heparinized saline then 4% paraformaldehyde (PFA). Brains were removed and fixed in 4% PFA for 48 h, then cryoprotected in 20% sucrose for 48 h, embedded in optimal cutting temperature (OCT) compound and frozen at − 80 °C. Frozen brains were sectioned into 10 μm slices.

Sections were washed in PBS, PBS + 0.1% triton x-100 (PBST), and PBS + 1% triton x-100. An antigen retrieval procedure was conducted where slides were placed in citric acid buffer and microwaved in a container of boiling water at full power for 5 min and at 1/10 power for 10 min, then allowed to cool to room temperature. Slides stained for BrdU were incubated with 2N HCl for 45 min at 37 °C, then washed again in PBST. Blocking was done in PBST + 5% normal donkey serum for one hour, and the primary antibody (rat-anti BrdU, Abcam, 1:200; rabbit-anti Olig2, EMD Millipore, 1:500; rabbit-anti Aldh1L1, Abcam, 1:500; mouse-anti NeuN, Abcam, 1:400) was incubated for 12 h overnight at 4 °C. The next day, following a wash in PBST, the secondary antibody (Alexa Fluor 488 donkey anti-rat, 1:500; Alexa Fluor 555 donkey anti-mouse, 1:500; Alexa Fluor 555 donkey anti-rabbit, 1:500) was incubated for 2 h at room temperature. Slides were then washed again in PBST and stained with DAPI nuclear stain for 5 min at room temperature before final washes and mounting. Slides were imaged using a Zeiss Axio Scope.A1 fluorescent microscope with AxioCam MRm camera and X-cite Xylis fluorescent module. Image brightness was adjusted using Adobe Photoshop CC 2018 to improve contrast from background to the same level for each antibody. For each stained slide, co-labelled cells were counted in three sections of the hypothalamus using the Photoshop count tool, then averaged to give a total for the slide. The researcher conducting cell counts was blinded to the experimental groups. Regions of interest were superimposed on the images throughout the hypothalamus to count co-labelled cells in specific nuclei, including the medial preoptic nucleus (MPO), suprachiasmatic nucleus (SCN), anterior hypothalamic nucleus (AHN), paraventricular nucleus (PVN), ventromedial hypothalamus (VMH), arcuate nucleus (ARC), tuberal nucleus (TU), dorsomedial hypothalamus (DMH), median eminence (ME), and lateral hypothalamus (LH).

### Statistical analysis

Data were analyzed using the GraphPad Prism software package. All data were tested to confirm normality using a Shapiro–Wilk normality test (α = 0.05) prior to analysis. A two-way ANOVA test was used to analyze postnatal weight, with Tukey post-hoc test if significant main effects were determined. Postnatal weight data for males and females was analyzed separately except for weights during adolescence (P5-P25) when males and females were indistinguishable. A one-way ANOVA was used to analyze cell counts of neurogenesis and gliogenesis with Sidak’s post-hoc test for multiple comparisons run between CON-STND vs. BPA-STND and CON-HFD vs. BPA-HFD on males and females separately. No animals or data points were excluded from analyses. A significance level of α = 0.05 was used throughout. All data are displayed as means ± SEM.

## Data Availability

The datasets used and/or analysed during the current study are available from the corresponding author on reasonable request.
